# Genome sequence of *Microbacterium foliorum* phage CandC

**DOI:** 10.1128/mra.01117-23

**Published:** 2024-01-17

**Authors:** Joëlle C. Makhoul, Megan Valentine, Caila Campbell, Emma G. McLaughlin, Frank H. Vereline, Jenna M. Collins, Sophia I. G. Crandall, Emma L. Rabideau, Wesley J. Tender, Shaniah L. Fairweather, Carson J. Miller, Kori Q. Y. Mcleish, Justin D. Izquierdo, Leah N. Gallagher, Luke P. Tyrrell, Alyssa M. Gleichsner

**Affiliations:** 1 Department of Biological Sciences, State University of New York, College at Plattsburgh, Plattsburgh, New York, USA; DOE Joint Genome Institute, Berkeley, California, USA

**Keywords:** bacteriophage assembly, genomics, assembly, virus, recombination

## Abstract

We report the discovery and genome sequence of CandC, a lytic bacteriophage with siphovirus morphology. CandC was isolated from a soil sample from Plattsburgh, NY, USA (Fall 2021). It has a genome size of 62,344 bp with 106 predicted protein-encoding genes, 30 of which are assigned putative functions.

## ANNOUNCEMENT

Actinobacteriophages are a diverse group of viruses known for their genetic mosaicism (1, 2). Bacteriophage genome characterization increases our understanding of co-evolutionary dynamics (3) and contributes to phage therapy applications (4). We present the genome of CandC, a bacteriophage collected in 2021 from Plattsburgh, NY (GPS coordinates 44.693117,–73.463150), that uses the bacterial host *Microbacterium foliorum* NRRL B-24224.

**Fig 1:**
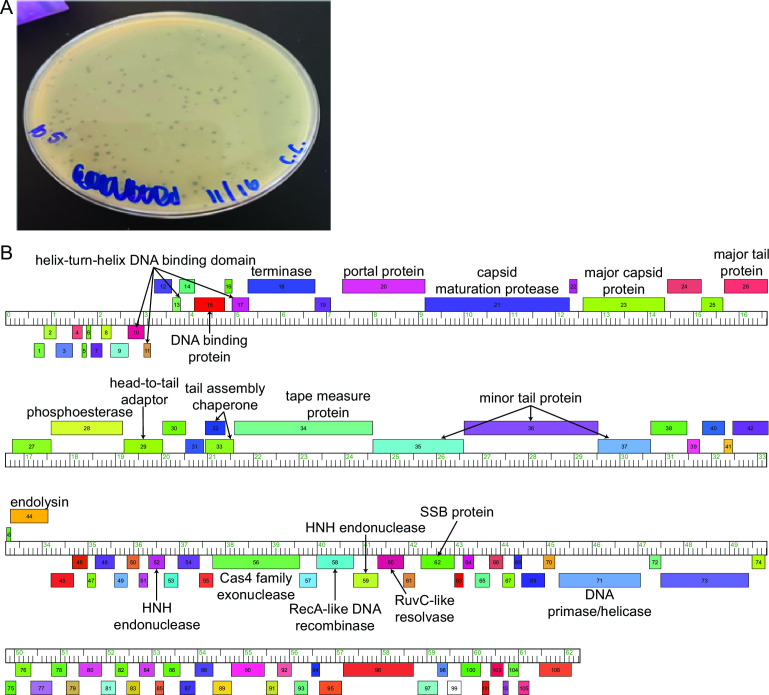
**(A**) Plaques of phage CandC in top agar with *M. foliorum*. (**B**) Genome annotation for CandC.

Following standard procedures (5), approximately 15 cm^3^ of soil was suspended in 35 mL PYCa medium and shaken at 37°C at 250 rpm for 2 h followed by centrifugation at 2,000 *g* and vacuum filtration (0.22 µm filter) of the supernatant. This filtrate was inoculated with *M. foliorum* and incubated at 30°C for 5 days at 250 rpm. An aliquot was spun at 14,000 *g*, filtered, and plated in PYCa top agar containing *M. foliorum*. After 48 h, CandC formed clear plaques with defined edges ~2 mm in diameter (Fig. 1A) and was purified through two rounds of plating.

DNA was extracted from a lysate (Wizard DNA Clean-up kit, Promega), prepared for sequencing (NEBNext Ultrall FS kit), and sequenced (Illumina sequencing, v3 reagents) resulting in 424,297 single-end 150-bp reads with 1,010-fold coverage. As described by Russell (6), the genome was assembled (Newbler v2.9) (7) and checked for completeness and genome termini (Consed v29.0) (8), resulting in a genome 62,344 bp in length with 203-bp direct terminal repeat ends and a GC content of 67%.

Using standard procedures (9), the software DNA Master v5.23.6 (http://cobamide2.bio.pitt.edu), PECAAN (https://discover.kbrinsgd.org), Genemark v2.5p (10), and Glimmer v3.02 (11) were used to automatically predict 109 putative protein-encoding genes. Start sites were determined using Starterator v485 (https://seaphages.org/software/#Starterator) and Blastp v2.13.0 (12) alignments against the Actinobacteriophage protein (13) and NCBI non-redundant protein sequences databases (https://blast.ncbi.nlm.nih.gov). No strong evidence for tRNAs was found using Aragornv1.2.41 (14) and tRNAscan-SE v2.0 (15). A total of 26 genes were assigned putative functions using BLASTp v2.13.0 (12), Phamerator (16), and HHPRED (searching against PDB_mmCIF70, SCOPe70, Pfam-A, and NCBI_Conserved_Domains databases) (17). deepTMHMM v1.0.24 (18) and SOSUI (19) detected four membrane proteins. All software used default settings. The annotation is presented in Fig. 1B.

Using the gene content similarity (GCS) tool at the Actinobacteriophage database (13) and based on GCS of at least 35% of phages in the database, CandC is assigned to the EG cluster (20). To date, there are 21 putative proteins unique to this cluster and shared by all its members, including several DNA-binding proteins, HNH endonuclease, DNA primase/helicase, tail assembly chaperones, and tape measure protein. Notably, EG cluster phages have a capsid maturation protease that is fused to a MuF-like protein, a major tail protein upstream of its head-to-tail connector protein (16, 20) and no identified integrase or repressor functions. CandC contains a single gene (#99) potentially acquired through a host-phage recombination event (21), as a search against the NCBI non-redundant database returns only a match with 56% amino acid identity to a hypothetical protein encoded by *Salmonella enterica* (GenBank accession No. WP_260797737.1).

## Data Availability

CandC is available at GenBank under the accession No. OR613466.1 and a Sequence Read Archive (SRA) of SRX22366550.
